# The high prevalence of intestinal parasitic infections is associated with stunting among children aged 6–59 months in Boricha Woreda, Southern Ethiopia: a cross-sectional study

**DOI:** 10.1186/s12889-020-09377-y

**Published:** 2020-08-20

**Authors:** Amanuel Yoseph, Hunachew Beyene

**Affiliations:** 1 Department of Public Health, College of Medicine and Health Science, Hawassa University, P.O. Box, 05, Hawassa, Ethiopia; 2 Department of Environment Health, College of Medicine and Health Science, Hawassa University, Hawassa, Ethiopia

**Keywords:** Children, Intestinal parasitic infection, Intestinal helminths, Under-nutrition and Ethiopia

## Abstract

**Background:**

Prior studies reported controversial results about the association between intestinal parasitic infections and childhood under-nutrition. We investigated the association of intestinal parasitic infections with under-nutrition among children aged 6–59 months in Boricha Woreda, Southern Ethiopia.

**Methods:**

This community-based prospective cross-sectional study was carried out from January 1–30, 2019 among 622 children aged 6–59 months. A two-stage stratified sampling procedure was used. Data were collected using a structured, face-to-face interviewer-administered questionnaire and standard anthropometric measurements. The stool specimens were collected using standard technique and examined for the existence and species of intestinal parasites using direct wet mount, Kato Katz and staining technique. We have entered data using Epi Data 3.1 and WHO Anthro software and all analyses were conducted using SPSS version 20. The descriptive analyses were done to find descriptive measures for the socio-demographic and other important variables. Multivariable logistic regression analysis was used to identify factors associated with under-nutrition. Adjusted odds ratios (AORs) with a 95% confidence interval (CI) were computed to assess the presence and strength of associations.

**Results:**

The total prevalence of intestinal parasitic infection was 48.7% (95% CI, 44.77–52.62). Approximately one-fourth (22%) of the children were infected with moderate intensity infections. Prevalence of stunting, underweight, wasting were 39.3, 24 and 11.6%, respectively. The prevalence of stunting among children infected with the intestinal parasite (59.4%) was significantly higher than the prevalence in non-infected children (20.6%) (*p* < 0.001). The absence of sanitation facility, living in medium and large family size, lack of shoes wearing practice, consuming raw vegetables and fruits were positively associated with intestinal parasitic infections. The presence of intestinal parasitic infections was positively associated with stunting (AOR = 2.18, 95% CI: 1.36–3.50) but not with wasting (AOR = 0.58, 95% CI: 0.3–1.13) and underweight (AOR: 0.92, 95% CI = 0.55–1.54).

**Conclusions:**

Under-nutrition and intestinal parasitic infections were serious public health concerns. Consolidating the prevailing water, sanitation and hygiene packages and routine deworming of children aged 6–59 months may aid to decrease the burden of both stunting and intestinal parasitic infection in children. Also, improving modern contraceptive methods utilization to reduce family size is recommended.

## Background

The term malnutrition generally refers to both under-nutrition and over-nutrition and anthropometric indicators that are commonly used to measure under-nutrition in a population are stunting, wasting and underweight [1].

Worldwide, the prevalence of under-nutrition is great. Its consequence in terms of public health is alarming. According to the World Health Organization (WHO) report, it was estimated that 178 million children had undernourished, 20 million children were suffering from the most severe form of under-nutrition and 3.5–5 million annual deaths occurred among children aged 6–59 months [2]. The economic cost of stunting is significant which accounts for a total loss of 10% disability-adjusted life years (DALYs) [3]. It has also resulted in the loss of adult height by 3%. Subsequently, childhood stunting might end up with 1.4% losses in productivity [4].

Under-nutrition among children is an outcome of many interrelated factors: socio-demographic, culture, environmental and food insecurity. Among these, inadequate dietary intake and infections have an immediate and direct effect [5]. An estimated half of under-nutrition was caused by repeated intestinal parasitic infections which have happened because of lack of safe or improved drinking water, inadequate sanitation, poor personal hygiene and environmental sanitation in Ethiopia [6].

According to the WHO report, about 24% (1.5 billion) of the world people have been infected with intestinal parasitic infections mainly hookworms, Ascaris *lumbricoides* and Trichuris *trichiura* [7]. The prevalence of intestinal parasitic infection surpasses 50% in several areas of sub-Saharan Africa (SSA) [7]. According to the 2015 assessment, the intestinal parasite has serious public health importance in the country (Ethiopia) [8]. In 2016, the nationwide prevalence among under-five children was 39% and the number did not decrease over the preceding period [9].

Intestinal parasitic infections inhabit the gastrointestinal tract (GIT) where nutrients are digested and absorbed and consequently results in a decline in food intake and/or an increase in nutrient wastage through blood loss, vomiting, inflammation-induced impairing of digestion and absorption, or diarrhoea. Hence, these effects can lead to or aggravate protein-energy malnutrition, anaemia and other nutrient deficiencies [10, 11].

WHO recommends annual medicinal treatment (deworming) in areas where the prevalence rate of soil-transmitted helminthiases is between 20 and 50%, and a bi-annual treatment in areas with prevalence rates of over 50% without a previous individual diagnosis to all at-risk people living in endemic areas. This intervention decreases morbidity by dropping the worm burden. Besides, health and hygiene education decrease transmission and reinfection by enhancing healthy behaviours, and the provision of acceptable sanitation is also vital but not always possible in resource-limited settings [7].

The worldwide long period aim is to eliminate morbidity and mortality related to intestinal parasitic infections among preschool and schoolchildren by 2020 [12]. Based on the Woreda administration report, intestinal parasitic infection hits the residents of almost all kebeles (the lowest administrative unit of Ethiopia with an approximate 1000 households) of the Woreda, and therapeutic and protective actions such as deworming school-age children using Albendazole or Membedazole were performed two times per year regularly. They are government and partner sponsored [13].

The relationship between intestinal parasitic infection and anaemia in children is well proven and systematic reviews have shown that deworming following a confirmed infection results in a substantial increase in haemoglobin in children [11, 14, 15].

However, the contribution of intestinal parasitic infection to under-nutrition in children has not been exhaustively explored and the prevailing evidence is controversial. Several observational studies carried out in Enemorena-Ener Woreda, Southern Ethiopia [16], rural Malaysia [15], Wakiso Woreda of central Uganda [10], Egypt [17] and Brazil [18] testified positive relationships. On the other hand, the studies carried out in Adama town, Ethiopia [19] and Northern Rwanda found no significant association [20]. Furthermore, according to a meta-analysis and observational study, a single dose of antihelminthic in the children showed no effect on the weight or height status of children [21, 22].

Thus, this study aimed to assess the relationship between intestinal parasitic infections and under-nutrition among children aged 6–59 months in Boricha Woreda, Southern Ethiopia.

## Methods

### Study area

The study was conducted in Boricha Woreda of Sidama regional state, Southern Ethiopia. The Woreda is located 305 km from Addis Ababa, the capital of the country. It is also 32 km from Hawassa, the capital of Sidama regional state. Based on the central statistical agency report of Ethiopia, the population of the Woreda was projected to be 450, 260 (4.16% urban and 93.84% rural). Of these, 13.94% were children in the age group of 6–59 months. The Woreda has consisted of 04 urban and 39 rural Kebeles. The elevation of the Woreda extends between 1501 to 2265 m above sea level and categorized into dual agro-ecological regions. The Lowlands share for 65% of the inhabitants while Midlands share for the left 35% of the residents. Based on the time of year, temperature differs from 15 to 35 °C and the average rainfall is 900 mm per year. The highest rainy period is ranged in early June and late October. The physical health service coverage of the Woreda was 90%. The Woreda has merely 1-government primary hospital, 10 health centres, 39 health post and 8 private clinics. The most common type of toilet facility in both urban and rural households was a pit latrine without a slab or open pit [13]. Farming is the major source of income-generating activity. The main crops grown in the area are enset (*false banana*), barely, khat, broad beans, cereals, and coffee. Unimproved drinking water source, poor hygienic condition and frequent drought in the Woreda results in a high burden of the under-nutrition and intestinal parasites [23]. Poor hygiene-related diseases, like acute watery diarrhoea and intestinal parasites, are among the leading causes of childhood morbidity and mortality [13].

### Study design and population

This community-based prospective cross-sectional study was conducted from January 1–30, 2019. The source and study population were all children in the age group of 6–59 months with their caregivers and all systematically selected children in the age group of 6–59 months with caregivers who resided in the Woreda for 6 months, respectively. The children whose parents/caregivers resided less than 6 months in the Woreda, children who had serious diseases, and treated last 1 month before the survey for any illness were excluded from the study.

### Sample size determination

The adequate sample size was estimated using a single population proportion formula in Epi Info TM 7 statistical package with the inputs of the proportion (p) of intestinal parasites (52.3%) was received from a previous study [24], 95% confidence level and 5% margin of error. As a two-stage sampling method was utilized to select the study children, a design effect of 1.5 was accounted, and a 10% compensation for non-response rate was considered. Thus, the final calculated sample size was 634.

### Sampling technique

A two-stage stratified sampling method was utilized to select representative study participants of this study. Firstly, we utilized a simple random sampling method to select representative kebeles from the Woreda. Secondly, we have utilized a systematic sampling method to select the study subjects. In Woreda, there are 43 Kebeles and eight kebeles were selected by using a simple random sampling method. Households (HHs) with 6–59 months aged children were distinguished by the house-to-house census and sampling frame which comprised of lists of HH in the selected kebeles was prepared. The total number of children aged 6–59 months who were eligible for the study were 8482 in the selected kebeles. Initially, the total sample size was allocated to the kebeles proportional to their population size. The calculated sample interval (K = N/n) was determined to be 14. Finally, the study children were selected using a systematic sampling strategy with a sampling interval of 14. The first child was selected by using a simple random sampling technique. Then, consecutive children were selected at a regular interval of the 14th HH until the needed sample size obtained. If a child was lacking from the HH for three sequential visits and there were no other alternatives, the next adjacent child was included. One child was included by using simple random sampling method when a twin or more than one child age group between 6 and 59 months found in the selected HHs. All children were provided with a unique identifier to be recognized during the stool examination and anthropometric measurements.

### Study variables

The outcome variables were nutritional status and intestinal parasitic infection. The independent variables were socio-demographic variables such as age, sex, religion, ethnicity, family size, wealth status, maternal or paternal education status, occupation status, marital status and media availability and accessibility; environmental factors such as the source of drinking water, types of sanitation facility, availability of solid waste disposal site, types of house and numbers of separate rooms; health-seeking behaviour such as personal hygiene practices, the technique of water treatment, handwashing practices, playing with soil, practice of eating raw fruits and vegetables and shoes wearing status; health service availability; dietary diversity score of the children and HH food insecurity status.

### Measurements

The data collection was run by 8 diploma nurses, 2 laboratory technicians and 8 community health worker data collectors. Two health officers carefully supervised the data collection procedure. Dietary diversity of the children was evaluated using the children’s Dietary Diversity Score tool (questions) of the Food and Agriculture Organization (FAO). The parents/caregivers were asked whether the children ate from seven standard food groups in the preceding day of the survey without putting the smallest consumption limits. Finally, dietary diversity score (DDS) was calculated out of the total score of seven and classified into inadequate or low (< 3) and adequate or high (> 4) [25]. The mid-upper arm circumference (MUAC) was measured following standard methods. MUAC indicators less than 11 cm indicates Severe Acute Malnutrition (SAM), between 11 and 12.5 cm indicates Moderate Acute Malnutrition (MAM), between 12.5–13.5 cm indicates mild malnutrition and above 13.5 cm indicates that the child is well nourished [1].

Food insecurity status was assessed by asking parents/caregivers from nine occurrences and frequency question of food insecurity. Based on the household food insecurity access prevalence (HFIAP) indicator, the households were categorized into four levels of household food insecurity (access): foods secure, mild, moderately and severely food insecure. Households are categorized as increasingly food insecure as they respond affirmatively to more severe conditions and/or experience those conditions more frequently [26].

All parents/caregivers were instructed by health extension workers (HEWs) to bring their children to the health posts. First, the parents/caregivers were told to bring about small fresh stool specimen of the child on the spot. In the process of stool examination, a simple and standard microscope (40X) known as Olympus was used. The stool samples were observed microscopically for the existence of eggs, trophozoites or cysts by using the direct wet mount, Kato Katz and staining technique to diagnose potential intestinal parasites. Infection intensity was estimated by averaging eggs per gram (epg) of faeces on both slides [27]. Finally, two laboratory technicians read each prepared slides, both of them blind for recheck purpose.

The anthropometric data of children were collected by using the measurement of age, height/length and weight. Height was measured using a measuring board and weight was measured using a SECA scale by appropriately trained nurses. Data collection was conducted in a stepwise manner in each kebele in their respective schedule. The weight of each child was taken with minimal clothing, without shoes, and with empty pockets. Measurement of height was done without shoes; to the nearest 0.1 cm. The raw anthropometric data of the studied children were converted to nutritional indicators using WHO Anthro Software (WFH) or Z-score by considering sex.

### Operational definitions

#### Intestinal parasitic infection is positive

Direct microscopic evidence of one or more parasites.

#### Stunting (chronic malnutrition)

Means HFA is below − 2 SD of the reference population while below − 3 SD indicates severe stunting.

#### Underweight (mixed malnutrition)

Means WFA is below − 2 SD of the reference population while below − 3 SD indicates severe underweight.

#### Wasting (acute malnutrition)

Means WFH is below − 2 SD of the reference population while below − 3 SD indicates severe wasting.

#### Family size

Is defined as an overall number of family members existing in the HH. Family size is categorized as small when it is < 6. It is categorized as a medium when it is ranged 6–8, and it is categorized as large when it is > 8.

### Data quality control

The data were collected using a structured, face-to-face interviewer-administered questionnaire and standard anthropometric measurements **(**Supplementary file 1). Initially, the study tool (questionnaire) was prepared in English language. Then, it was converted into Sidamic language. Lastly, it was retranslated back to English to retain its accuracy and consistency. The evaluation was conducted to consider the consistency and accuracy of the two types of study tool. The pre-test was conducted on 5% of samples in kebeles other than actual study area. At that time, any inconsistency and non-accuracy were adjusted so. The data of the pretest analysis was not used for the final sample of our study. The training was provided for data collectors and supervisors by the principal investigators for 2 days. The training was aimed at the study objective, methods and data collection procedure. Moreover, regular checkup for incompleteness, non-accuracy and inconsistency of the data were made daily.

### Data analysis

Data were entered into Epi Data 3.1 and WHO Anthro software and analyzed using SPSS version 20. All needed variables recording and calculations were carried out earlier to the major analysis. The descriptive analyses were done to find descriptive measures for the socio-demographic and other important variables. The chi-square(X^2^) test was utilized to describe the association between independent and dependent variables. A cross-tabulation was utilized to assess the main assumption of chi-square. Sensitivity analysis was carried out to consider the influence of missing data by using multiple imputation methods. Principal Component Analysis (PCA) was conducted in the calculation of the wealth index. Wealth index was computed as a composite indicator of living standard based on 25 variables associated to possession of carefully chosen household assets, the scale of agricultural land, number of livestock, supplies utilized for house building, and ownership of improved water and sanitation facilities. The analysis generated a summary score that explained 65.1% of the variability of the data and the score was finally ranked into five categories such as lowest, second-lowest, middle, second-highest and highest.

Bi-variable and multivariable binary logistic regression analyses were used to identify factors of under-nutrition. The bi-variable logistic regression analysis started with unadjusted analysis in which every possible factor was evaluated independently for its relationship with under-nutrition. The variables with *p*-values < 0.2 on the bi-variable logistic regression analysis were entered into a multivariable model to get independent factors of under-nutrition controlling for other factors in the model [28]. The major assumptions of the binary logistic regression model such as the absence of influential cases, multicollinearity and interaction between independent variables were tested to be fulfilled. Consequently, no one of the interaction terms was statistically significant showing nonexistence of significant effect modification. The multicollinearity among the independent variables was also evaluated using a multiple linear regression model. For all variables the tolerance statistics was,> 0.1 and variance inflation factor (VIF) was < 10 which indicates no evidence of multicollinearity. The fitness of the binary logistic regression model was also assessed in the model using the Hosmer-Lemeshow statistic (0.85). The existence and strength of association among independent variables and under-nutrition were evaluated using adjusted odds ratios (AORs) with a 95% CIs. A statistically significant association was confirmed when the 95% CI of the (AORs) did not contain 1.

## Results

Socio-demographic and economic features of the study respondents have been summarized in Table 1. From a total of 634 sample size, merely 622 study respondents answered questions, making a response rate of 98.2%. The mean (+standard deviation [SD]) age of children was 27 + 18 months. The mean family size of each HH was 6 persons. The majority, 596 (95.8%) and 517 (83.1%) of the study respondents were from Sidama ethnic group and followers of protestant Christianity, respectively. The majority, 505 (81.2%) of the household’s income was obtained from farming activity. Two-third of the mothers had never attended formal education. Almost all 614 (98.7%) of the HHs were headed by fathers. Nearly 249 (40%) of families had access to social media such as radio and television. Protected water sources were the main source of drinking water for 485 (78%) of the studied household participants. The majority, 420 (67.5%) of the study HHs had pit latrines without a slab/open pit. It was also observed that 251 (40.4%) of the studied children did not wear shoes. The majority, 408 (65.8%) of the studied household used a single room for sleeping purpose.

**Table 1:** Socio-demographic and economic characteristics of the study respondents in Boricha Woreda, South Ethiopia, 2019 (*N* = 622)

Variables	N (%)
Age in months	
6–11	123 (19.8)
12–23	146 (23.47)
24–59	353 (56.73)
Sex	
Male	305 (49)
Female	317 (51)
Ethnic group	
Sidama	596 (95.8)
Others	26 (4.2)
Religions of respondents	
Protestant	517 (83.1)
Others	105 (16.9)
Family size	
Small (< 6 members)	300 (48.2)
Medium (6–8 members)	117 (18.8)
Large (> 8 members)	205 (33)
Maternal education status	
No formal education	401 (64.4)
Primary education (1–8 grade)	159 (25.6)
Secondary education & above	62 (10)
Paternal education status	
No formal education	394 (63.3)
Primary education (1–8 grade)	122 (19.6)
Secondary education & above	106 (17.1)
Main occupation of study respondents	
Farmer	506 (81.2)
Others	116 (18.8)
Wealth status	
Lowest	154 (24.8)
Second lowest	72 (11.6)
Middle	157 (25.2)
Second highest	117 (18.8)
Highest	122 (19.6)
The major source of drinking water	
Protected	485 (78)
Unprotected	137 (22)
Availability of sanitation facility	
Yes	449 (72.2)
No	173 (27.8)
Types of sanitation facility	
Pit latrine without a slab	420 (67.5)
Pit latrine with slab	29 (4.7)
Number of rooms used for sleeping purpose	
One	408 (65.8)
Two	214 (34.2)
Hand washing practice	
Yes	394 (63.3)
No	228 (36.7)
Shoes wearing practice	
Yes	371 (59.6)
No	251 (40.4)
The practice of consuming raw vegetables and fruits	
Yes	184 (29.6)
No	438 (70.4)

### Dietary diversity score and nutritional status of children

The general quality of the diet of the children was also evaluated according to the diet consumed in the preceding day of the survey. Grains, roots or tubers (66.6%) and dairy products (62.5%) were consumed by the majority of the study children. Less frequently consumed food groups were: eggs (44.2%), Vitamin-A rich fruits and vegetables (34.60%), flesh foods (25.4%), legumes and nuts (21.50%) and other fruits and vegetables (12.7%).

The mean (± SD) dietary diversity score was 3.67 (± 1.07). Merely 39.7% consumed from 4 or more food groups indicative of adequate dietary diversity. On the other hand, 60.3% had low dietary diversity score (less than 3 food groups).

The mean (± SD) MUAC of the children was 14.67 (±1.44) cm. More than one-tenth (12.5%) of the children had MUAC between 11 and 12.5 cm indicative of moderate acute malnutrition. Only 3.4% of the children had mild acute malnutrition (MUAC range 12.5–13.5 cm). None of the children had severe acute malnutrition. Only 51% of parents/caregivers reported that the children took the deworming tablet at least once in the preceding 6 months of the survey. Likewise, 90.4% of parents/caregivers reported that the children took vitamin A supplementation.

### The prevalence of under-nutrition

Prevalence of stunting, underweight, wasting were 39.3, 24 and 11.6%, respectively. Among these, 3.4, 1.8 and 2.1% of the children had severely stunted, underweighted and wasted, respectively. Stunting was commonest under-nutrition problem (Table 2).

**Table 2:** Nutritional status of study children aged 6–59 months in Boricha Woreda, Southern Ethiopia, 2019 (*N* = 622)

Variables	N (%)
Height for age	
Below −3 SD (Severe)	21 (3.4)
Below −2 SD (Stunted)	223 (35.9)
Weight for age	
Below −3 SD (severe)	11 (1.8)
Below −2 SD (Under-weight)	138 (22.2)
Weight for height	
Below −3 SD (Severe)	13 (2.1)
Below −2 SD (Wasting)	59 (9.5)

### The prevalence of intestinal parasitic infections

The prevalence of at least one intestinal parasitic infection was 48.7% (95% CI = 44.77–52.62). The prevalence of each intestinal parasite was as follows: *Giardia lamblia (*10.45%) and Entamoeba *histoltica (*4.66%). Similarly, the prevalence of intestinal helminthic parasites included the following species: Ascaris *lumbricoides (*10.77%), Hookworm (7.88%), Trichiura *trichiura* (6.1%), Strangloild *stercoralis* (1.6%) and Taenia *species (*1.3%). Infection with more than one intestinal parasite (mixed infections) was 5.94% among children in the current study. Heavy infection with any species was seen in 7.6% of the children (Table 3).

**Table 3:** The prevalence of intestinal parasitic infections of study children aged 6–59 months in Boricha Woreda, Southern Ethiopia, 2019 (*N* = 622)

Variables	Sex		Total	*p*-value
	Male (%)	Female (%)		
Types of infections				
Single	128 (20.58)	138 (22.19)	266 (42.77)	0.964
Mixed	18 (2.89)	19 (3.05)	37 (5.94)	0.755
Types of intestinal parasites				
*Giardia lamblia*	20 (3.22)	45 (7.23)	65 (10.45)	0.002**
Entamoeba *histoltica*	14 (2.25)	15 (2.41)	29 (4.66)	0.998
Ascaris *lumbricoides*	26 (4.18)	41 (6.59)	67 (10.77)	0.049*
Trichiuris *trichiuria*	27 (4.34)	11 (1.76)	38 (6.1)	0.001**
Hookworm	33 (5.3)	16 (2.58)	49 (7.88)	0.008*
Taenia *species*	2 (0.32)	6 (0.96)	8 (1.3)	0.054*
Strongliod *stercoralis*	6 (0.96)	4 (0.64)	10 (1.6)	0.097
Intensity of infection				
Light	101 (16.24)	112 (18)	213 (34.24)	0.625
Moderate	32 (5.14)	35 (5.62)	67 (10.77)	0.978
Heavy	12 (1.93)	11 (1.77)	23 (3.7)	0.899

*: Shows significant association at *p*-value < 0.05 **: Show the highly significant association at *p*-value < 0.01

**Fig. 1:**
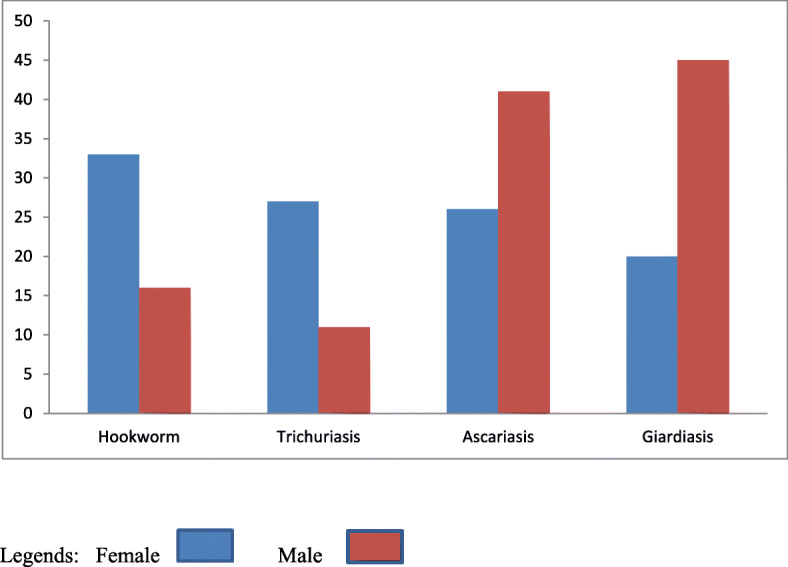
Prevalence of intestinal parasitic infections by sex in Boricha Woreda among children aged 6–59 months, Southern Ethiopia, 2019

The three common parasitic infections such as Ascaris *lumbricoides*, hookworm and Trichiuris *trichiura* collectively called soil-transmitted infections and two common intestinal parasitic infections *Giardia lamblia* and Entamoeba *histoltica* are called a protozoan. Among them, the prevalence of *Giardia lamblia* and Ascaris *lumbricoides* were higher in females than males. However, Trichiuris *trichiura* and hookworm were higher in male (Fig. 1).

### Predictors of intestinal parasitic infections

Findings of the bi-variable and multivariable logistic regression analyses of intestinal parasites are presented in Table 4. Both bi-variable and multivariable logistic analyses of predictors showed that odds of intestinal parasitic infections were 2.7 times increased in children living in large family size as compared to those living in small family size (AOR = 2.70; 95% CI = 1.50–5.00; *P* = 0.001). The absence of sanitation facility (AOR = 2.9; 95% CI = 1.60–5.30; *P* = 0.001) and lack of shoes wearing practice (AOR = 3.5; 95% CI =2.20–5.70; *P* = 0.001) were positively associated with intestinal parasitic infections. The odds of intestinal parasitic infections were 2.65 times increased for children who had a habit of consuming raw vegetables and fruits (AOR = 2.65, 95 CI = 1.60–4.70) as compared to those who had no habit of consuming raw vegetables and fruits.

**Table 4:** Bi-variable and multivariable logistic regression analysis of predictors of intestinal parasitic infections among children aged 6–59 months in Boricha Woreda, Southern Ethiopia, 2019 (*N* = 622)

Variables	Intestinal Parasitic infections		COR	AOR
	Yes (%)	No (%)		
Sex of child				
Male	146 (47.86)	159 (52.14)	1	1
Female	157 (49.53)	160 (50.47)	1.06(1.01,1.46)	0.65(0.12,1.02)
Wealth status				
Lowest	113 (73.38)	41 (26.62)	5.65(3.35,9.5)	1.04(0.49,2.22)
Second lowest	39 (54.16)	33 (45.83)	2.42(1.33,4.4)	0.79(0.34,1.78)
Middle	69 (43.94)	88 (56.06)	1.6(0.98,2.62)	0.57(0.29,1.10)
Second highest	42 (35.90)	75 (74.10)	1.14(0.67,1.95)	0.57(0.28,1.15)
Highest	40 (32.79)	82 (67.21)	1	1
The major source of drinking water				
Protected	199 (41.03)	286 (58.97)	1	1
Unprotected	104 (75.91)	33 (24.09)	4.52(3.13,7.62)	1.14(0.58,2.24)
Diarrhoea past two week				
Yes	53 (51.96)	49 (48.04)	1.17(0.76,1.78)	0.72(0.24,1.04)
No	250 (48.08)	270 (51.92)	1	
The practice of consuming raw vegetables & fruits				
Yes	143 (77.72)	41 (22.28)	6(4.07,9.02)*	2.65(1.6,4.7)*
No	160 (36.53)	278 (63.47)	1	1
Absence of sanitation facility				
Yes	135 (78.03)	38 (21.94)	5.9(4,8.9)*	2.9(1.6,5.3)**
No	168 (37.41)	281 (62.59)	1	1
Wearing Shoes				
Yes	127 (34.23)	244 (65.77)	1	1
No	176 (70.12)	75 (29.88)	4.5(3.2,6.4)*	3.5(2.2,5.7)**
Family size				
Small	78 (26)	222 (74)	1	1
Medium	71 (60.68)	46 (39.32)	4.4(2.8,6.9)*	2.3(1.3,4.2)*
Large family	154 (75.12)	51 (24.88)	8.6(5.7,12.9)*	2.7(1.5,5)**

1: Shows the reference categories

*: Shows significant association at *p*-value < 0.05 **: Show the highly significant association at *p*-value < 0.01

### Association between intestinal parasitic infections and under-nutrition

The prevalence of stunting among children infected with intestinal parasitic infections (59.4%) was considerably greater than the prevalence in non-infected children (20.6%) (*p* < 0.001). Approximately all of the children infected with Ascaris *lumbricoides* (92.53%) were stunted. Moreover, the prevalence of stunting was alarmingly great among children identified with other parasitic infections: Hookworm (89.79%), G *lamblia* (86.20%), E *histolytica* (72.24%), T *trichiura* (71%), Taenia *species* (50%) and Strongliod *stercoralis* (50%). The statistically significant relationship was identified between stunting and Hookworm, G *lamblia*, E *histolytica* and T *trichiura* (*p* < 0.05) (Table 5).

**Table 5:** The relationship between specific types of intestinal parasitic infections and stunting among children aged 6–59 months in Boricha Woreda, Southern Ethiopia, 2019 (*N* = 622)

Type of parasitic infection	Stunting		Prevalence of stunting (%)	(*p*-value)^x^
	Yes	No		
Any parasitic infection				
Yes	178	125	59.4	< 0.001**
No	66	253	20.6	
Giardia *lamblia*				
Yes	56	9	86.2	< 0.001**
No	188	369	33.75	
Entamoeba *histoltica*				
Yes	21	8	72.24	0.006*
No	223	370	37.6	
Ascaris *lumbricoides*				
Yes	62	5	92.53	< 0.001**
No	182	373	32.79	
Trichiuris *trichiuria*				
Yes	27	11	71.05	0.007*
No	217	367	37.15	
Hookworm				
Yes	44	5	89.79	< 0.001**
No	200	373	34.9	
Taenia *species*				
Yes	4	4	50	0.23
No	240	374	39	
Strongliod *stercoralis*				
Yes	5	5	50	0.22
No	239	373	39	

× Chi-square and fisher exact test used for the analysis of the variables

*: Shows the significant association at *p*-value < 0.05 **: Show the highly significant association at *p*-value < 0.01

The bi-variable regression model analysis indicated children who were infected with intestinal parasitic infection had 5.45 times increased odds of stunting (COR = 5.45, 95% CI: 3.82–7.78) as compared to non-infected children. In the multivariable logistic regression model in which 10 possible confounders were controlled, the odds of stunting were also 2.18 times increased (AOR = 2.18, 95% CI: 1.36–3.50) among children infected with intestinal parasitic infections (Table 6). The analysis of the complete bi-variable and multivariable logistic regression model is delivered as supplementary material **(**Supplementary file 2**).**

**Table 6:** The relationship between intestinal parasitic infections and stunting among children aged 6–59 months in Boricha Woreda, Southern Ethiopia, 2019 (*N* = 622)

Variable	Nutritional status		COR	AOR^a^
	Normal	Stunted		
Intestinal parasitic infections				
Yes	125	178	5.45(3.82,7.78)*	2.18(1.36,3.50)*
No	253	66	1	1

1: Shows the reference categories *: Shows the significant association at *p*-value < 0.05

^a^ Adjusted for sex, maternal and paternal educational status, the main occupation of the head, family size, wealth index, main source of drinking water, household food insecurity and dietary diversity score

However, both bi-variable and multivariable logistic regression model analysis of predictors for wasting and underweight revealed that the presence of intestinal parasitic infections had no contribution to low weight for height (AOR = 0.58, 95% CI: 0.3–1.13) and weight for age (AOR: 0.92, 95% CI = 0.55–1.54) status (Tables 7 and 8).

**Table 7:** Bi-variable and multivariable analysis of the predictors of underweight of children aged 6–59 months in Boricha Woreda, South Ethiopia, 2019 (*N* = 622)

Variables	Nutritional status		COR	AOR^a^
	Normal	Underweight		
Sex				
Male	240 (78.70)	65 (21.30)	0.75(0.51–1.08)*	0.58(0.38–0.89)**
Female	233 (73.50)	84 (26.5)	1	1
Food insecurity status				
Secure	279 (94.30)	17 (5.70)	1	1
Insecure	194 (59.50)	132 (40.5)	11.16(6.52,19.11)*	3.50(1.78,6.94)**
Presence of parasite				
Yes	198 (65.3)	105 (34.7)	3.31(2.22,4.92)	1.07 (0.65,1.77)
No	275 (86.2)	44 (13.8)	1	1
Dietary diversity score				
Adequate(> 4)	228 (92.30)	19 (7.70)	1	1
Low (< 3)	245 (65.33)	130 (34.77)	6.36 (2.51,9.84)*	5.79(2.55,13.13)*

1: Shows the reference categories *: Shows the significant association at *p*-value < 0.05

**: Show the highly significant association at *p*-value < 0.01

^a^ Adjusted for sex, maternal and paternal educational status, the main occupation of head, family size, wealth index, main source of drinking water, household food insecurity and dietary diversity score

**Table 8:** Bi-variable and multivariable analysis of the predictors of wasting of children aged 6–59 months in Boricha Woreda, South Ethiopia, 2019 (*N* = 622)

Variables	Nutritional status		COR	AOR^a^
	Normal	Wasted		
Dietary diversity score				
Adequate(> 4)	233 (91.91)	15 (8.09)	1	1
Low (< 3)	317 (86.13)	57 (13.87)	2.79 (2.05,6.34)*	2.22(1.33,5.79)*
Presence of intestinal parasite				
Yes	250 (82.50)	53 (17.50)	3.34(1.93,5.80)	0.57(0.29, 1.10)
No	300 (94.00)	19 (6.00)	1	1
Maternal education status				
No formal education	311 (84.05)	59 (15.95)	3.49(2.46,8.05)*	3.25(1.89,9.41)**
Have formal education	239 (93.36)	13 (6.64)	1	1

1: Shows the reference categories*: Show the significant association at *p*-value < 0.05 **: Show the highly significant association at *p*-value < 0.01

^a^ Adjusted for sex, maternal and paternal educational status, the main occupation of head, family size, wealth index, main source of drinking water, household food insecurity and dietary diversity score

## Discussion

The study indicated that almost half (48.7%) of children in Boricha Woreda have intestinal parasitic infections and mixed infection with polyparasites was also common. Moreover, the prevalence of stunting, underweight, wasting were 39.3, 24 and 11.6%, respectively suggestive of severe public health significance of under-nutrition in a population. After controlling for possible confounders, we identified a positive association between intestinal parasites and stunting.

The prevalence of under-nutrition reported in this study is suggestive of severe public health significance of under-nutrition in a population [1]. Several facility or community-based studies in elsewhere established the similar [9, 29–32]. The current EDHS reported 38, 24 and 10% of children in Ethiopia were stunted, underweighted and wasted, respectively [9]. The facility-based studies in Shashemene referral hospital, West Arsi Zone Ethiopia (38.3, 49.2 and 25.2%) [32]; Haramaya Woreda, Eastern Ethiopia (45.8, 21 and 10.7%) [31]; Bule Hora Woreda, Southern Ethiopia (47.6, 29.2 and 13%) [30]; and Hidabu Abote Woreda, North Shewa Ethiopia (47.6, 30.9 and 16.7%) [29], also reported public health significance statistics.

The prevalence of intestinal parasite infection was 48.7%, with the two leading prevalence for *Ascaris lumbricoides (*10.77%) and *Giardia lamblia (*10.45%) in this study. If the prevalence of intestinal parasitic infection was greater than or equal to 50%, it is classified as high in Ethiopia. Based on the national categorization of intestinal parasitic infection prevalence, the result of the recent study is reached to high classification [8]. Furthermore, it is far away from the short term national target decrease of the intestinal parasitic infections of less than 1% in Ethiopia. As a result, it is difficult to meet the national target of eliminating intestinal parasitic infection by 2020 [8, 12]. Similar studies conducted in Ethiopia reported varied figures and patterns, indicating the presence of a significant difference in the epidemiology of intestinal parasitic infection at the country level [24, 33–36].

A study carried out in Addis Ababa, Ethiopia reported an extremely high prevalence (71.8%) of infections with intestinal parasites, and distinguished A. *lumbricoides* (34.9%), T. *trichiuria* (22.8%) and *G. lamblia* (9.6%) as the major infections [33]. A cross-sectional study among children in North Shoa, Ethiopia (52.3%) reported an equivalent statistics to our result (52.3%) yet the predominant infection was *Giardia lamblia* (19.6%) [24]. A similar study carried out in a Hawassa Zaria Woreda, South Ethiopia, reported 51.3% overall prevalence and A. *lumbricoides* was the prevalent infection affecting 42.2% of the children [34]. A community-based study in Ethiopia estimated 24.3% total prevalence and, A. *lumbricoides* was most frequently faced parasitic infection (19%) [36]. A study in Woreta Health centre, Northwest Ethiopia found 18.7% prevalence of intestinal parasitic infections with the high proportion of *Giardia lamblia* (6.8%) followed by Entamoeba *histoltica* (3.4%) [35].

The prevalence of *Giardia lamblia* and Ascaris *lumbricoides* were higher in females than males children. This finding agreed with the study conducted in the selected village of Pawi special Woreda in Benishangul-Gumuz Region, Northwestern Ethiopia and Akwanga, central Nigeria [37, 38]. This might be because of the lack of providing adequate care to the female children and more specifically improper child handling practice in the rural part of the country (Ethiopia). Similarly, the prevalence of Trichiuris *trichiura* and hookworm were higher in males than females children. This is consistent with the study finding from Northern Bolivia Altiplano, Mohakhali in Dhaka city and South Thailand [39–41]. It needs a further study to identify what factors lead the sex difference in intestinal parasitic infections.

The odds of intestinal parasitic infections were 2.65 times increased for children who had a habit of consuming raw vegetables and fruits as compared to those who had no habit of consuming eat raw vegetables and fruits. This is in line with the study result from Thailand [42]. This might be because cleaning raw vegetables and fruits is an important aspect to reduce any opportunity for intestinal parasitic transmission.

The absence of sanitation facility in households was positively associated with intestinal parasitic infections. This result is in agreement with the studies conducted in Ethiopia [24, 43]. This might be the contamination of water and food with human waste due to defecation in the open field. Further, a lack of shoes wearing practice was positively associated with intestinal parasitic infections. This result agreed with the study carried out in Northwest Ethiopia [43]. This might be due to lack of awareness of parent and children on how intestinal parasites are transmitted to human and walking on barefoot instead of wearing shoes permitting penetration of the skin by the infective agents.

Multivariable analysis of predictors revealed that odds of intestinal parasitic infections were 2.7 times increased in children living in large family size as compared to those living in small family size. This is consistent with the study findings from Addis Ababa of Ethiopia [33]. This might be due to that overcrowded family use single room to sleep creates an amplified chance for intestinal parasitic transmission.

After adjusting for possible confounders including several socio-demographic status indicators, we identified a statistically significant association between intestinal parasitic infection and stunting. This is in agreement with a study done in North India [21]. Similarly, the finding from India and Uganda showed that a significant contribution of intestinal parasitic infection on the occurrence of the stunting [10, 44]. Moreover, a cross-sectional study conducted in Egypt reported that significantly lower weight for age Z-score was found among infested children as compared to non-infected ones [45]. As mentioned prior, the detected association might be clarified by many biological mechanisms. Intestinal parasitic infection can impair nutritional status by sucking nutrients from the intestinal mucosa or by enhancing chronic nutrients loss, decreasing appetite and nutrient consumption, competing for nutrition and causing diarrhoea or dysentery in children [11].

In our study, the contribution of the intestinal parasites for the development of underweight and wasting was insignificant. This may be the effect of a small sample size. The parameter estimates 1.07 is significant but not statistically because of the confidence interval CI = 0.65–1.77. This is consistent with a study conducted in North India [21]. In contrary to the current study, the finding from India, and Uganda showed a significant contribution of intestinal parasitic infection on the occurrence of the underweight and wasting [10, 44]. Moreover, a cross-sectional study conducted in Egypt reported that significantly lower weight for age and weight for height Z-score among infested children as compared to non-infected ones [45].

This result may suggest that in addition to the routine vitamin A supplementation (VAS), protective or curative deworming combined with Integrated Management of Neonatal and Childhood Illness (IMNCI) may benefit to decrease the problem of childhood stunting. As of public health viewpoints, this would provide more logic in the thought of the fact that only 51% of the children in this study received deworming tablets in the preceding 6 months of the survey. Based on the national guideline of the country (Ethiopia), children should be routinely dewormed in the six-monthly bases. However, a national study showed that in 2016 merely 26% of the children received deworming tablet [9].

### Limitation of the study

Our study had several strengths. Among these, the community-based nature of the current study is representative of all children aged 6–59 months and generates important evidence to develop an applicable policy strategy for effective elimination of intestinal parasitic infection and reduction of stunting. In the fact that we recruited in a comparatively large number of children (*n* = 622) from different kebeles and described prevalence statistics for different under-nutrition and intestinal parasites. Moreover, we tried to evaluate and accounted for several potential confounders that can individually describe the relationship between the variables of interest. Irrespective of its strengths, the current study had certain fundamental limitations that may be careful while inferring the results. First, due to the cross-sectional nature of the study design, it was challenging to precisely establish any possible temporary relationship between variables of interest. Second, this study may be liable to recall bias because some part of the information was collected by the parents/caregivers self-report. Third, children are only enrolled within a month, and there might be a seasonal fluctuation of the prevalence of under-nutrition and intestinal parasites. Earlier surveys have witnessed that the prevalence of several intestinal parasitic infections in the human population is liable to inter-seasonal variation [46, 47]. Fourth, similar to several other observational studies, we controlled for possible confounders using the multivariable logistic regression model. However, residual confounding/confounding from unevaluated sources (e.g. malaria, measles, HIV, urinary parasites including urinary schistosomiasis, and other co-morbidities) cannot be omitted. It is also significant to consider that the survey was merely restricted to intestinal parasitic infection and did not observe into the effect of malaria an essential hemiparasite in the study area. Finally, 51% of the study children received deworming medication in the last 6 months of the preceding survey, the study is probable to underestimate the expecting figure of the problem.

## Conclusions

This study showed that 48.7% of the children in Boricha Woreda have intestinal parasitic infection and 39.3, 24 and 11.6% were stunted, underweighted and wasted, respectively. The most prevalent species of intestinal parasitic infections were Ascaris *lumbricoides* and *Giardia lamblia*. Mixed infection with polyparasites was commonly identified. Furthermore, a positive association was identified between intestinal parasitic infections and stunting. Consolidating the prevailing water, sanitation and hygiene packages and routine deworming of children aged 6–59 months may aid to decrease the burden of both stunting and intestinal parasitic infection in children. Also, improving modern contraceptive methods utilization to reduce family size is recommended. The result of sex difference on the prevalence of the intestinal parasitic infections should be further investigated.

## Supplementary information

**Additional file 1: Supplementary file 1.** English version study tool (questionnaire).

**Additional file 2: Supplementary file 2.** Complete output of bi-variable and multivariable logistic regression analysis in Boricha Woreda, Southern Ethiopia, 2019.

## Data Availability

The datasets generated and/or analyzed during the current study are not publicly available due to institutional regulation but are available from the corresponding author on reasonable request.

## Abbreviations

AOR: Adjusted odds ratio
ASL: Above sea level
CI: Confidence Interval
DDS: Dietary diversity score
FAO: Food and agricultural organization
HFA: Height for age
HFIAP: Household Food Insecurity Access Prevalence
IMNCI: Integrated management of newborn and childhood illness
MAM: Moderate acute malnutrition
MUAC: Mid-upper arm circumference
OR: Odds ratios
PCA: Principal component analysis
SAM: Severe acute malnutrition
SD: Standard Deviation
SNNPR: South nations, nationalities and people region
SPSS: Statistical packages for social science
VAS: Vitamin A supplementation
VIF: Variance inflation factors
WFA: Weight for age
WFH: Weight for height
WHO: World Health Organization
