# Supplementary material for: The high prevalence of intestinal parasitic infections is associated with stunting among children aged 6–59 months in Boricha Woreda, Southern Ethiopia: a cross-sectional study
Source: BMC Public Health. 2020 Aug 20;20:1270. doi: 10.1186/s12889-020-09377-y (PMC7441631; doi:10.1186/s12889-020-09377-y)
Supplement: Supplementary file 1 — Additional file 1: Supplementary file 1. English version study tool (questionnaire). [file 12889_2020_9377_MOESM1_ESM.docx]

# Annex I: English version questionnaire

**Hawassa University**

**College of Medicine and Health Sciences**

**School of Public Health**

***Questionnaire designed to study the intestinal parasitic infections and its association with under-nutrition among children aged 6-59 months in Boricha district, Southern Ethiopia***

**1. Information sheet**

Hello! My name is _------------------------------------------------------_. I am a data collector in a survey being conducted about intestinal parasitic infections and under-nutrition under the College of Medicine and Health Sciences of Hawassa University. The aim of this study is to determine the association between intestinal parasitic infection and under-nutrition among children aged 6-59 months in Boricha district, Southern Ethiopia. The purpose of this study is to generate information about the intestinal parasitic infections and under-nutrition among children in Boricha district. The study may help stakeholders, policy makers, responsible body, and others to take actions based on the finding. The study comprises various intimate and private questions and few physical measurements such as weight, height, MUAC and stool samples. You are chosen to participate in this study by chance. Interview and physical measurements will take not more than 30 minutes.

We assure you that there is no risk or harm in participation of this study. All information will be kept confidentially. Name of a participant will not be written or specified. Your privacy will also be protected and no one shall know your response.

This study benefits you that, you have the right to know physical measurement findings and status of intestinal parasatosis and undernutrion of your child. There is no incentive or payment for participating in this research. Likewise, findings of the study will show the magnitude and severity of the problem among study population. This in turn will help to design effective and appropriate measure for prevention and control of intestinal parasitic infections and under-nutrition.

You have full right whether or not to participate in this study. You may respond to all questions or you may not answer to the questions you don’t want to or you may quit your participation totally at any time you want. You can ask any questions which is not clear for you.

**2. Informed consent**

As to the information given ahead, participating in this study has no risk. In order to attain the objective of the study, your participation is vital. For this reason we are requesting your free will. You are selected randomly to participate in this study and your name will not be written on this form and the information you give will never be shared to others. Your genuine response to the interviews will be very important for the purpose of the study. You have a right to refuse in responding any question or the entire question at any time you want.

I have read this form or it has been read to me in the language I comprehend and understand all condition stated above.

Are you willing to participate in this study?

Yes □ No □

If “Yes" ...proceed with the interview.

If “No” ...........thank you and end.

Name of the principal investigator: Amanuel Yoseph

Cell phone Number - +251-915683578 E mail – amanuelyosep45@gmail.com

Name of interviewer___________________ Signature__________

Data of interviewer (Ethiopia calendar) _____/_____/_____

Result of interview: 1. Complete 2. Refused 3. Partially complete 4. Respondent not available

Cheeked by supervisor: Name______________ Signature_________ Data___/_____/____

**Part I: Socioeconomic and Demographic characteristics**

| S. n | Questions | | Responses | | skip |  |
| --- | --- | --- | --- | --- | --- | --- |
| 1 | Your child’s sex? | | Male…….. Female……. | |  |  |
| 2 | How old is your child? | | …………….. | |  |  |
| 3 | What is your ethnicity? | | 1. Sidama 2. Wolayita 3. Amahara 4. Other (specify)……………. | |  |  |
| 4 | What is your religion? | | 1. Protestant 2. Muslim 3. Orthodox 4. Catholic 5. Other(Specify) | |  |  |
| 5 | Who is the head of HH? | | 1. Father 2. Mather 3. Relative 4. Other(Specify) | |  |  |
| 6 | The main occupation of head of HH? | | 1. Farmer 2. Merchant 3. Government employer 4. Unemployed 5. Other(Specify) | |  |  |
| 7 | Paternal educational status? | | 1. No formal education 2. Read and write only 3. Primary education completed 4. Secondary education completed 5. Other(Specify) | |  |  |
| 8 | Mother educational status? | | 1. No formal education 2. Read and write only 3. Primary education completed 4. Secondary education completed 5. Other(Specify) | |  |  |
| 9 | How many family members do you have? | | ………… | |  |  |
| 10 | Do you have access to electricity? | | 1. Yes b) No | |  |  |
| 11 | Do you have television? | | a)Yes b) No | |  |  |
| 12 | Do you have radio? | | a)Yes b) No | |  |  |
| 13 | Do you have windows? | | a)Yes b) No | |  |  |
| 14 | What is the roofing material (for the main house) made from? | | 1. Grass roof 2. Corrugated iron roof 3. Wood and mud 4. Mud and stone 5. Brick tiles 6. Other(Specify) | |  |  |
| 15 | What is the floor material (for the main house) made from? | | 1. Mud 2. Cement 3. Wooden 4. Bricks 5. Other(Specify) | |  |  |
| 16 | What is the wall material (for the main house) made from? | | 1. Wood and mud 2. Stone and mud 3. Stone and cement 4. Hallow blocks 5. Bricks 6. Other(Specify) | |  |  |
| 17 | How many rooms do you have? | | ................................ | |  |  |
| 18 | How many rooms in this household are used for  Sleeping? | | ……………………. | |  |  |
| 19 | How many birr you earn monthly? | | ……………………. | |  |  |
| 20 | | Does this household have all domestic animals? | | 1. Yes 2. No |  | |
| 21 | | Do you have the following domestic animals? | | 1. Cow 2. Ox 3. Donkey/hourse/mule 4. Sheep 5. Goat 6. Hen 7. Honey hive 8. Others (Specify)………. |  | |
| 22 | | Do you have farm land? | | 1. Yes 2. No |  | |
| 23 | | How many hectare of land do you have? | | ………………………. |  | |
| 24 | | Do you have bank book for any member of households? | | 1. Yes 2. No |  | |
| 25 | | Does this household have the following material? | | 1= Yes 2= no   1. Watch…………1 2 2. Mobile………….1 2 3. Bicycle……………1 2 4. Moter bicycle………1 2 5. Hoarse/donkey cart…..1 2 6. car…………………….1 2 7. Moter Bajaj……………..1 2 8. Others (specify)………….1 2 |  | |

**Part II: Water and Sanitation**

| 26 | Main source of drinking water for your family? | 1. Tap/public 2. Unprotected well/spring 3. Protected well/spring 4. River, stream/lake/dam 5. Other(Specify) |  |
| --- | --- | --- | --- |
| 27 | If your water source is other than pipe, how long does it take to fetch water? Get water and come back | 1. Less than an hour 2. One hour or more 3. Water on premises 4. Other(Specify) |  |
| 28 | Do you treat your water in any way to make it safer to drink? (do not included washing water container) | 1. Yes, always 2. Yes, sometimes 3. No, |  |
| 29 | If your drinking water is other than pipe, what do you do to improve its quality? (more than one answer is possible) | 1. No 2. Boil 3. Strain it through a cloth 4. wuha agar 5. Let it stand and settle 6. Use water filter(sand, composite) 7. Water purifying product 8. Other(Specify) |  |
| 30 | How much of water you used per households per day? | ……………………….L |  |
| 31 | Do you have toilet facility? | a)Yes b) No |  |
| 32 | If yes to question no 26, What kind of toilet facility does your HH use? | 1. Pit /latrine without slap/open pit 2. Pit /latrine with slap 3. VIP 4. Flash latrine 5. Other(Specify) |  |
| 33 | How often the families use the latrine? | 1. Always 2. Some times 3. Never use |  |
| 34 | How many HHs share /use the same toilet? | 1. One 2. Two to Three 3. Four to nine 4. Ten or more |  |
| 35 | How does family dispose your house waste? | 1. Burning 2. In to waste pit 3. Open field 4. Other specify |  |
| 36 | Does your child always wash his hands, before he eat? | 1. Yes, always 2. Yes, sometimes 3. No |  |
| 37 | When your child washes his hands, what substance does he use for hand washing? | 1. None (only with water) 2. Soap (shampoo) 3. Sand 4. Ash 5. Plant extract 6. Other(specify) |  |
| 38 | If the child uses soap, how often he use the detergent while washing his hands? | ………… |  |
| 39 | Does your child brush his teeth before eating breakfast today? | 1. Yes always 2. Some times 3. No |  |
| 40 | How often do you wash your hands after using the toilet or latrine? | 1. No 2. Always 3. Some times |  |
| 41 | How often your child does baths his body? | 1. Every days 2. Every other day 3. Every week 4. once in fifteen days 5. Once month 6. Once two month 7. Other(specify |  |
| 42 | Does your child eat raw vegetables and fruits? | 1. Yes 2. No |  |
| 43 | Does your child always wear shoes? | 1. Yes 2. No |  |

**Part III: Household food insecurity access scale questions**

| 1 | In the past four weeks, did you worry that your HH would not have enough food? | 0= No……………………...  1=Yes | Q2 |
| --- | --- | --- | --- |
| 1.a. | How often did this happen? | 1=Rarely  2= Sometimes  3= Often |  |
| 2 | In past four weeks, were you or any HH member not able to eat the kinds of foods you preferred B/c of a lack of resources? | 0=No……………………..  1=Yes | Q3 |
| 2.a. | How often did this happen? | 1=Rarely  2= Sometimes  3= Often |  |
| 3 | In past four weeks, did you or any HH member have to eat a limited variety of foods B/c of a lack of resources? | 0=No………………………..  1=Yes | Q4 |
| 3.a. | How often did this happen? | 1=Rarely  2= Sometimes  3= Often |  |
| 4 | In past four weeks, did you or any HH member have to eat some food that really did not want to eat B/c of a lack of resources to obtain other types of food? | 0= No……………………..  1=Yes | Q5 |
| 4.a. | How often did this happen? | 1=Rarely  2= Sometimes  3= Often |  |
| 5 | In past four weeks, did you or any HH member have to eat a smaller meal than you felt you needed B/c there was not enough food? | 0=No………………………  1=Yes | Q6 |
| 5.a. | How often did this happen? | 1=Rarely  2= Sometimes  3= Often |  |
| 6 | In past four weeks, did you or any HH member have to eat a fewer meals in a day’s B/c there was not enough food? | 0=No………………………  1=Yes | Q7 |
| 6.a. | How often did this happen? | 1=Rarely  2= Sometimes  3= Often |  |
| 7 | In past four weeks, was there ever no food to eat of any kind in your HH B/c of lack of resource to get food? | 0=No………………………..  1=Yes | Q8 |
| 7.a | How often did this happen? | 1=Rarely  2= Sometimes  3= Often |  |
| 8 | In past four weeks, did you or any HH member go to sleep at night hungry B/c there was not enough food? | 0=No………………………..  1=Yes | Q9 |
| 8.a. | How often did this happen? | 1=Rarely  2= Sometimes  3= Often |  |
| 9 | In past four weeks, did you or any HH member go to a whole day and night without eating anything B/c there was not enough food? | 0=No………………………..  1=Yes | Q |
| 9.a. | How often did this happen? | 1=Rarely  2= Sometimes  3= Often |  |

**Part IV: Access health service and health seeking behavior**

| 44 | Did you visit the health facility? | 1. Yes b) No |  |
| --- | --- | --- | --- |
| 45 | If no to Question number 40, why? | 1. Too far 2. Too costly 3. I don’t know it 4. My family never met an illness symptoms |  |
| 46 | Has the child had an illness with diarrhea in the last two weeks? | 1. Yes b) No |  |
| 47 | If yes, did you seek advice or treatment for the illness outside home? | 1. Yes b) No |  |
| 48 | From where did you seek care? Anywhere else? | 1. Modern medical care center 2. Traditional healers 3. Spiritual healers 4. Holy water 5. Other (specify) |  |
| 49 | Does your child have received deworming tablets in the last six months? | 1. Yes 2. No |  |
| 50 | Does your child have received vitamin A supplementation in the last six months? | 1. Yes 2. No |  |

**Part V: Dietary diversity questionnaire**

Instruction: Please describe the foods or drink that you took yesterday during the day or at night.

| S.NO | Questions and filters | Coding categories |
| --- | --- | --- |
| 1 | Now I would like to ask you about foods that your child ate yesterday during the day or at night. |  |
|  | Place a 1/one/ in the box. If she/he ate the food or drink in question, place a zero/0/ in the box if she/he not eaten the food in question. |  |
| A | Grains, roots or tubers | A …………………………………… /……/ |
| B | Legumes and nuts | B………………………………………/…../ |
| C | Dairy products(milk, yogurt, cheese) | C …………………………………… /……/ |
| D | Flesh foods (meat ,fish, poultry and liver/organ meat | D ……………………………………/…. / |
| E | Eggs | E………………………………………/…../ |
| F | Vitamin-A rich fruits and vegetables | F ……………………………………../…../ |
| G | Other fruits and vegetables | G………………………………………/…../ |

Anthropometric measurement

Now I would like to take your child height and weight

Weight in KG

|  |  |  |  |  |  |
| --- | --- | --- | --- | --- | --- |

Height in CM

|  |  |  | . |  |  |
| --- | --- | --- | --- | --- | --- |

Thank you very much for your cooperation!!
