# Supplementary material for: The high prevalence of intestinal parasitic infections is associated with stunting among children aged 6–59 months in Boricha Woreda, Southern Ethiopia: a cross-sectional study
Source: BMC Public Health. 2020 Aug 20;20:1270. doi: 10.1186/s12889-020-09377-y (PMC7441631; doi:10.1186/s12889-020-09377-y)
Supplement: Supplementary file 2 — Additional file 2: Supplementary file 2. Complete output of bi-variable and multivariable logistic regression analysis in Boricha Woreda, Southern Ethiopia, 2019. [file 12889_2020_9377_MOESM2_ESM.docx]

**Table 6:** Association between intestinal parasitosis and stunting among children aged 6-59 months in Boricha Woreda, Southern Ethiopia, 2019 (N=622)

| Variables | Nutritional status | | COR | AOR |
| --- | --- | --- | --- | --- |
|  | Normal (%) | Stunted (%) |  |  |
| Intestinal parasitic infection |  |  |  |  |
| Yes | 125 (41.30) | 178 (58.70) | 5.45 (3.82,7.78)* | 2.18 (1.36,3.50)* |
| No | 253 (79.30) | 66 (20.70) | 1 | 1 |
| Sex |  |  |  |  |
| Male | 164 (53.77) | 141 (46.23) | 1.78 (1.29,2.47)* | 1.90 (1.27,2.86)** |
| Female | 214 (67.50) | 103 (32.50) | 1 | 1 |
| Food insecurity status |  |  |  |  |
| Secure | 259 (87.50) | 37 (12.50) | 1 | 1 |
| Insecure | 119 (36.50) | 207 (63.50) | 12.17 (8.06,18.37)* | 4.36 (2.49,7.64)** |
| Dietary diversity score |  |  |  |  |
| Adequate(>4) | 223 (90.30) | 24 (9.70) | 1 | 1 |
| Low (<3) | 155 (41.30) | 220 (58.7) | 13.18 (8.25,21.07)* | 3.59 (2.01,6.41)* |
| Family size |  |  |  |  |
| Small(1-5 members) | 232 (77.30) | 68 (22.70) | 1 | 1 |
| Medium(6-7 members) | 61 (52.10) | 56 (47.90) | 3.13 (1.99,4.92) | 0.98 (0.54,1.81) |
| Large(>8 members) | 85 (41.50) | 120 (58.5) | 4.18 (3.26, 7.09) | 1.06 (0.59, 1.90) |
| Wealth index |  |  |  |  |
| Lowest | 67 (43.50) | 87 (56.50) | 4.56 (2.68,7.78) | 1.23 (0.62, 2.47) |
| Second lowest | 39 (54.20) | 33 (45.80) | 2.97 (1.58, 5.59) | 1.16 (0.53, 2.56) |
| Middle | 88 (56.10) | 69 (43.90) | 2.75 (1.62, 4.69) | 1.97 (1.01, 3.87) |
| Second highest | 89 (76.10) | 28 (23.90) | 1.10 (0.60, 2.02) | 0.80 (0.38, 1.69) |
| Highest | 95 (77.90) | 27 (22.10) | 1 | 1 |
| Maternal education status |  |  |  |  |
| No formal education | 168 (45.90) | 198 (54.10) | 5.38 (3.68, 7.86) | 1.47 (0.44, 4.90) |
| Have formal education | 210 (82.00) | 46 (18.00) | 1 | 1 |
| Paternal education status |  |  |  |  |
| No formal education | 215 (80.20) | 53 (19.80) | 4.75 (3.29, 6.85) | 0.87 (0.27, 2.81) |
| Have formal education | 163 (46.00) | 191 (54.00) | 1 | 1 |
| The main occupation of the head |  |  |  |  |
| Farmer | 284 (56.10) | 222 (43.90) | 3.34 (2.03, 5.48) | 1.27 (0.68, 2.38) |
| Others | 94 (81.00) | 22 (19.00) | 1 | 1 |
| The main source of drinking water |  |  |  |  |
| Protected | 325 (67.00) | 160 (33.00) | 3.21 (2.17, 4.76) | 0.86 (0.51, 1.44) |
| Unprotected | 53 (38.70) | 84 (61.30) | 1 | 1 |

1: Indicates the reference categories

*: Indicates the significant association (P-value < 0.05) **: Indicate the highly significant association (P-value <0.01)
